# Patients' experience and satisfaction using telemedicine for outpatient services in a Tertiary Cancer Center in Qatar during COVID‐19: A cross‐sectional study

**DOI:** 10.1002/hsr2.883

**Published:** 2022-10-29

**Authors:** Israa Elhakeem, Phool Iqbal, Abdulqadir J. Nashwan, Muhammad Abubakar, Arsalan T. Jawad, Mousa A. AlHiyari, Prem Chandra, Maab A. Osman, Sara S. Mohamad, Mohammed Alkhatib, Mohamed A. Yassin

**Affiliations:** ^1^ Medical Education Hamad Medical Corporation Doha Qatar; ^2^ Hematology/Oncology Department Hamad Medical Corporation Doha Qatar; ^3^ Internal Medicine Department New York Medical College/Metropolitan Hospital Center New York USA; ^4^ Nursing Department Hamad Medical Corporation Doha Qatar; ^5^ Academic Health System Hamad Medical Corporation Doha Qatar; ^6^ Medical Critical Care Department Hamad Medical Corporation Qatar

**Keywords:** cancer, COVID‐19, teleconsultations, telehealth, telemedicine

## Abstract

**Background and Aim:**

The coronavirus‐19 is an ongoing global pandemic resulting in millions of deaths worldwide. For a patient population at higher risk of infection, telemedicine is a promising means of providing safe and alternative care routes while minimizing their risk of exposure. This study gives insight into patients' experiences and satisfaction with telemedicine during this pandemic.

**Methods:**

We conducted a cross‐sectional study on 297 patients (RR: 85%) at the National Center for Cancer Care and Research (NCCCR), Qatar. Data was collected through electronic medical records of the eligibe patient population, and phone calls were made whereby the physician read a standard introductory script followed by a survey questionnaire. We focused on patients' experience with telemedicine services amid the pandemic. This was done using a six‐point Likert scoring system of seven questions that were scaled from 1 to 6.

**Results:**

More than 80% of patients somewhat to strongly agreed that telemedicine met their healthcare needs, improved their confidence in their healthcare system, and were generally satisfied with the quality of care provided. Nearly all patients (90%) understood their physicians' recommendations over the phone. In addition, more than half of the patients (89%) felt they could freely communicate their concerns. Patients also showed an inclination towards face‐to‐face consultations at 68%; however, 90% were willing to participate in future teleconsultations.

**Conclusion:**

Our study indicates an overall positive experience among patients towards the use of telemedicine. Telemedicine is a safe, futuristic approach toward patient care management and, thus, provides healthcare professionals a platform to implement further patient and physician education. Even though our data also showed that patients liked in‐person visits to some degree, this needs to be looked into more in future studies.

## 1 INTRODUCTION

The world health organization (WHO) declared Coronavirus 19 (COVID‐19) a global pandemic in March 2020.^1^ As of November 2021, there has been over 240 million cases worldwide and over 5 million deaths.^2^ Although it has caused a burden on hospitals worldwide, it also gave an insight to the pre‐existing gaps within the structure of health care systems.^3^ Due to global shortage of hospital necessities such as personal protective equipment, healthcare workers were more susceptible to contracting the virus.^4^ WHO has estimated that as of May 2021, at least 80,000 to 180,000 healthcare workers worldwide died due COVID‐19.^5^ Controlling it's spread has proven a challenge, considering asymptomatic individuals can also transmit the virus.^6^ There is also a great possibility that asymptomatic affected healthcare workers amplify the spread of the virus to nonexposed patients.^7^ As a consequence, hospitals worldwide were overburdened, resulting in delaying services such as outpatient appointments and elective procedures.^8^ Cancer care was not an exception, many countries postponed routine cancer screening programs, and patients with diagnosed cancers experienced interruptions in their anticancer treatments.^9^, ^10^ Telemedicine provides an alternative and safer way to receive care while minimizing the risk of exposure to the virus.^11^ It is the use of an electronic communication to exchange medical information.^12^ This is done through a growing variety of services, such as audio or video calls, emails, and other forms of telecommunication^13^, ^14^ proving to be convenient for both patients and physicians. As a result, there has been an increase in the use of telemedicine during this pandemic, which has been shown to decrease costs and visit timing and result in high patient satisfaction.

However, there are also many obstacles faced with implementing it, such as technological illiteracy among populations without digital access, and language barriers.^15^ Previous publications have illustrated the drawbacks of telemedicine including a systematic review which found that the top barriers were related to technology specific challenges, they suggested these could be overcome by training and change in management techniques.^16^ Another more recent systematic review showed that common patient barriers included low technology literacy, lack of technological resources, lack of trust in technology and perceived ethical and security concerns. It also showed that there was lack of evidence in using telemedicine in the prevention and surveillance of recurrence as well as new cancers.^17^

Another important consideration is the question of feasibility of telemedicine in low‐income regions and elderly population. A Retrospective cohort study found that lower income areas as well as adults aged 85 years or older were more likely to use phone visits over video visits, however lack of digital and cellular access proved to be a common challenge.^18^ Practicality in the developing world is also questionable. A recent review of telemedicine in Sub‐Saharan Africa demonstrates clear barriers including insufficient technological infrastrucutre and medical equipment, digital illiteracy, inaccessibility to medical care and financial barriers with lack of adquate funding.^19^ Table 1 including a summary of the list of studies exploring the role of telemedicine during COVID‐19.

**Table 1:** The list of studies exploring the role of telemedicine during COVID‐19

Title	DOI	Type of Study	country	Sample	Conclusion
Patient and physician attitudes toward telemedicine in cancer clinics following the COVID‐19 pandemic^20^	10.1200/CCI.20.00183	A question‐based survey	United states of America	1843	Mixed results, the discrepancy between patient and provider concern for spread of infectious disease represents an area where patients may benefit from increased education.
Telemedicine during the COVID‐19 pandemic: Impact on care for rare cancers^21^	10.1200/GO.20.00220	Questionnaire based survey of clinicians and patients	London, United Kingdom	283	Telemedicine can revolutionize delivery of cancer care, particularly for patients with rare cancers who often live far away from expert centres
Rapid implementation of telemedicine during the COVID‐19 pandemic: Perspectives and preferences of patients with cancer^22^	10.1002/onco.13676	Interview based on survey questionnaire	Israel	172	Telemedicine is perceived as safe and effective, and often does not compromise medical care or the patient‐physician relationship
Physicians' perceptions on the role of telemedicine in cancer care during and post‐COVID‐19 pandemic^23^	10.47895/AMP.V55I2.2836	Online questionnaire survey	Philippines	84	Telemedicine was perceived by Filipino physicians in a tertiary hospital as an acceptable solution for the provision of cancer care during and after the COVID‐19 pandemic
Providing supportive and palliative care using telemedicine for patients with advanced cancer during the COVID‐19 pandemic in Mexico^24^	10.1002/onco.13568	Feedback after providing telemedicine interventions	Mexico	45	Supportive tool for low‐ and middle‐income countries and maintain continuity of care while decreasing the spread of virus.
Patients'/caregivers' perspectives on telemedicine service for advanced cancer patients during the COVID‐19 pandemic: An exploratory survey^25^	10.4103/IJPC.IJPC_145_20	Exploratory survey based on	India	NA	Telemedicine is an important tool and an essential service to care for palliative care patients in the community
Patient and provider‐reported satisfaction of cancer rehabilitation telemedicine visits during the COVID‐19 pandemic^26^	10.1002/pmrj.12552	Prospective survey study	United States	184	data support that telemedicine visits should be considered essential as part of comprehensive cancer rehabilitation care
Analysis of the implementation of telehealth visits for care of patients with cancer in Houston during the COVID‐19 pandemic^27^	10.1200/OP.20.00572	Qualitative study	United States	1762	Oncology/hematology patients and their physicians expressed high levels of satisfaction with the use of telehealth video visits
Telemedicine in the context of Covid‐19—A qualitative study of cancer patients and clinicians^28^	10.21203/rs.3.rs-149380/v1	Qualitative study	Australia	48	Role of telemedicine in the follow up of MO patients seems likely to continue beyond Covid‐19.
Abstract SS2‐09: Telemedicine usability for cancer care during the COVID‐19 pandemic^29^	10.1158/1538-7445.sabcs20-ss2-09	Qualitative study	United States	132	The utility of telemedicine in cancer care during the COVID‐19 pandemic was perceived favorably by both patients and providers
Telemedicine usability for cancer care during the COVID‐19 pandemic^30^	10.1200/JCO.2020.38.29_suppl.265	Qualitative study	United States	105	The use of telemedicine in cancer care was perceived favorably by patients and providers
Using telemedicine during the COVID‐19 pandemic: Attitudes of adult health care consumers in Israel^31^	10.1002/jso.26327	Observational study	Israel	693	Monitoring patients' attitudes regarding telemedicine is essential in the future after the pandemic ends. Telemedicine is effective.
Teleoncology or telemedicine for oncology patients during the COVID‐19 pandemic: The new normal for breast cancer survivors?^32^	10.2217/fon-2020-0714	Descriptive cross‐sectional study	NA	135	The study showed that telemedicine could open a new era for medical oncology specialists.
Oncologist perspectives on telemedicine for patients with cancer: A National Comprehensive Cancer Network Survey^33^	10.1200/op.21.00195	Survey study	NA	1038	Substantial fraction of visits for patients with cancer could be effectively and safely conducted using telemedicine. These findings should influence regulatory and infrastructural decisions regarding telemedicine post pandemic for patients with cancer.
Utility of telemedicine in sub‐Saharan Africa during the COVID‐19 pandemic. A rapid review^19^	10.1002/hbe2.297	Rapid systemic review	Within Sub‐Sahran Africa	NA	Suggested that telemedicine was an appropriate intervention for continuation of health services in a pandemic. However, challenges such as lack of lack of mobile devices, internet connection, health insurance impended the spread of service.
Impact of COVID‐19 on cancer care delivery in Africa: A Cross sectional survey of oncology providers in Africa^34^	10.1200/GO.20.00569	Cross sectional Survey	Zambia, Nigeria, Botswana, Kenya, Zimbabwe, Ethiopia, Mozambique, Ghana, South Africa, Republic of Congo, Cabo Verde, Cameroon, Burkina Faso. Egypt, Namibia, Sudan. Tanzania	79	For those who used telehealth majority rated it as favorable care of delivery however the challenge of internet connectivity remained a challenge. They could not comment on cost effectiveness.

The National Center for Cancer Care and Research is the primary cancer hospital in Qatar. It has utilized telemedicine to continue its services to its patients amid the pandemic. The objective of this qualitative analysis was to explore the experiences and satisfaction that cancer patients had with telemedicine during the pandemic. As telemedicine is new our cancer care service, we reviewed their experience to further advanced telemedicine for both hematology and oncology patients.

## 2 METHODS

We conducted a study in a tertiary cancer care center (NCCCR) in Qatar. We were planning to include a total of 400 participants, however, we managed to enroll 297. Our inclusion criteria included patients regularly followed by telemedicine in outpatient clinics during COVID 19, patients over 18 years of age, the ability to complete the questionnaire over the phone, and no language barriers. In addition, we excluded patients who did not respond to the phone call after five attempts (at different times on different days), patients who were abroad, patients who did not consent to answer the questionnaire, and patients who expired. Using this well‐formulated questionnaire, we analyzed the patient satisfaction scale and took further comments on telemedicine to improve the clinic's services during the COVID‐19 pandemic. Data was collected using phone calls with a standard introductory script that was read out by a team of physicians to the patients in their native language. A survey questionnaire was formulated to measure patients' feedback and satisfaction scales for improving telemedicine clinic services during the COVID‐19 pandemic. It was divided into two main categories; category 1 comprised patients' demographics, and category 2 comprised seven questions on their feedback and satisfaction scale. Each of the seven questions was scaled from one through six using a six‐point Likert scoring system. The phone calls last for not more than 10 min for each participant.

## 3 RESULTS

Over the course of 6 weeks, 297 patients were contacted over the phone to answer the survey questionnaire. Participants were found to be predominantly female (67%). The cohort of patients included 46.1% with solid tumor malignancy, 38.4% with benign hematological conditions, and 15.5% with hematological malignancies. Please refer to Table 2 for additional demographics.

**Table 2:** Demographic and Clinical Characteristics for Patients (*N* = 297)

Characteristics	Total number (percentage%)	
Gender		
Female	199	69%
Male	98	33%
Marital status		
Single (never legally married)	64	21.5%
Married/common law (two people living together but not married)	216	72.7%
Separated/divorced	6	2%
Widowed	11	3.7%
Work status		
Full time in the paid work force (30 h or more per week)	142	47.8%
Part time in the paid work force (less than 30 h per week)	5	1.7%
Self‐employed	3	1%
Unemployed	81	27.3%
Disability/sick leave	2	0.7%
Homemaker	37	44.4%
Retired	27	13.8%
Level of education		
Elementary School	23	7.7%
High School	81	27.3%
Technical or vocational school or preuniversity degree	20	6.7%
University (Undergraduate Bachelor)	132	44.4%
University (Graduate: Masters, Doctorate or Post Doctorate degree	41	13.8%
Diagnosis		
Solid tumor	137	46.1%
Hemtological malignancy	46	15.5%
Benign hematology	114	38.4%

**Figure 1:**
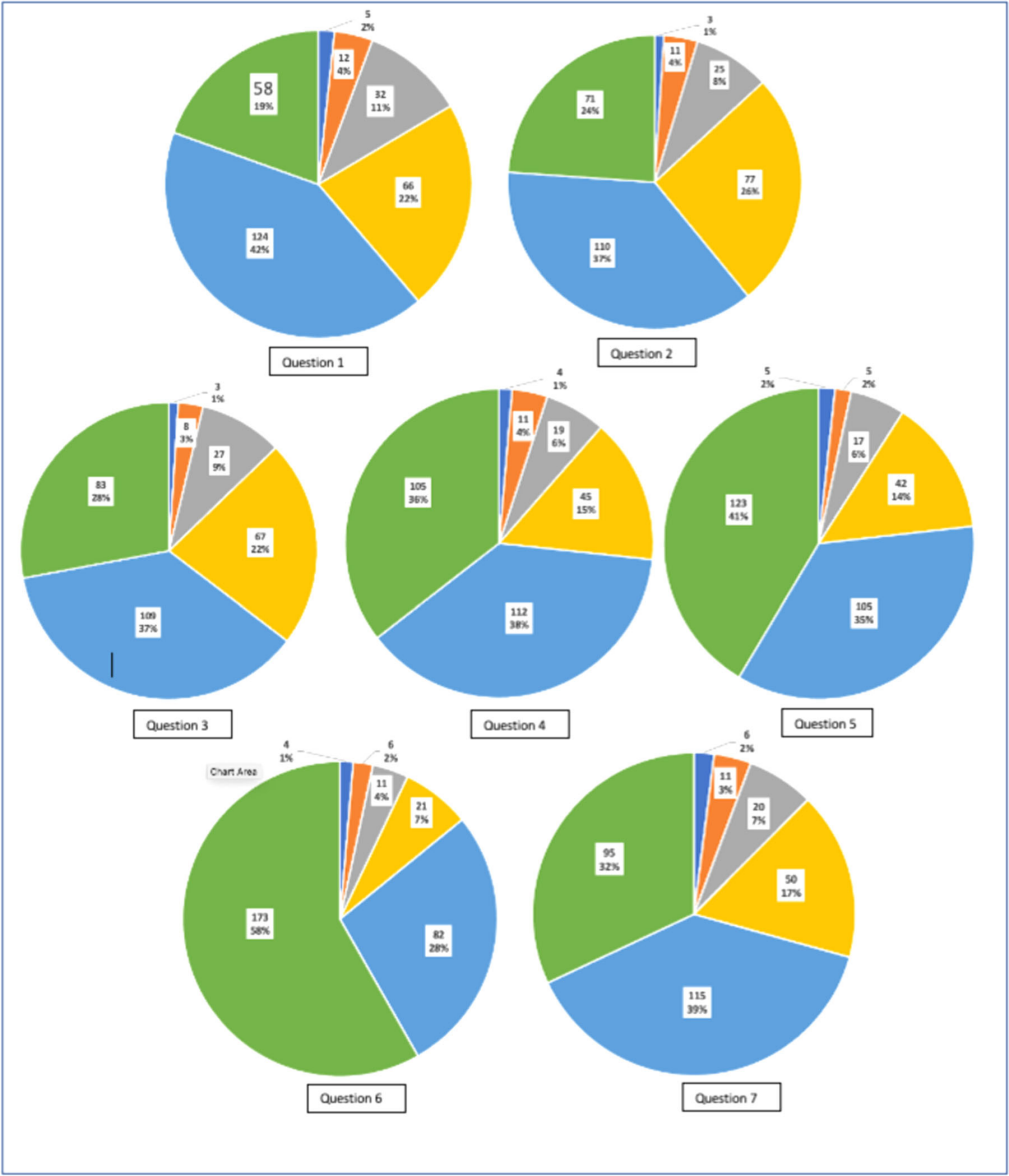
Patients feedback and satisfaction questions 1–7

**Figure 2:**
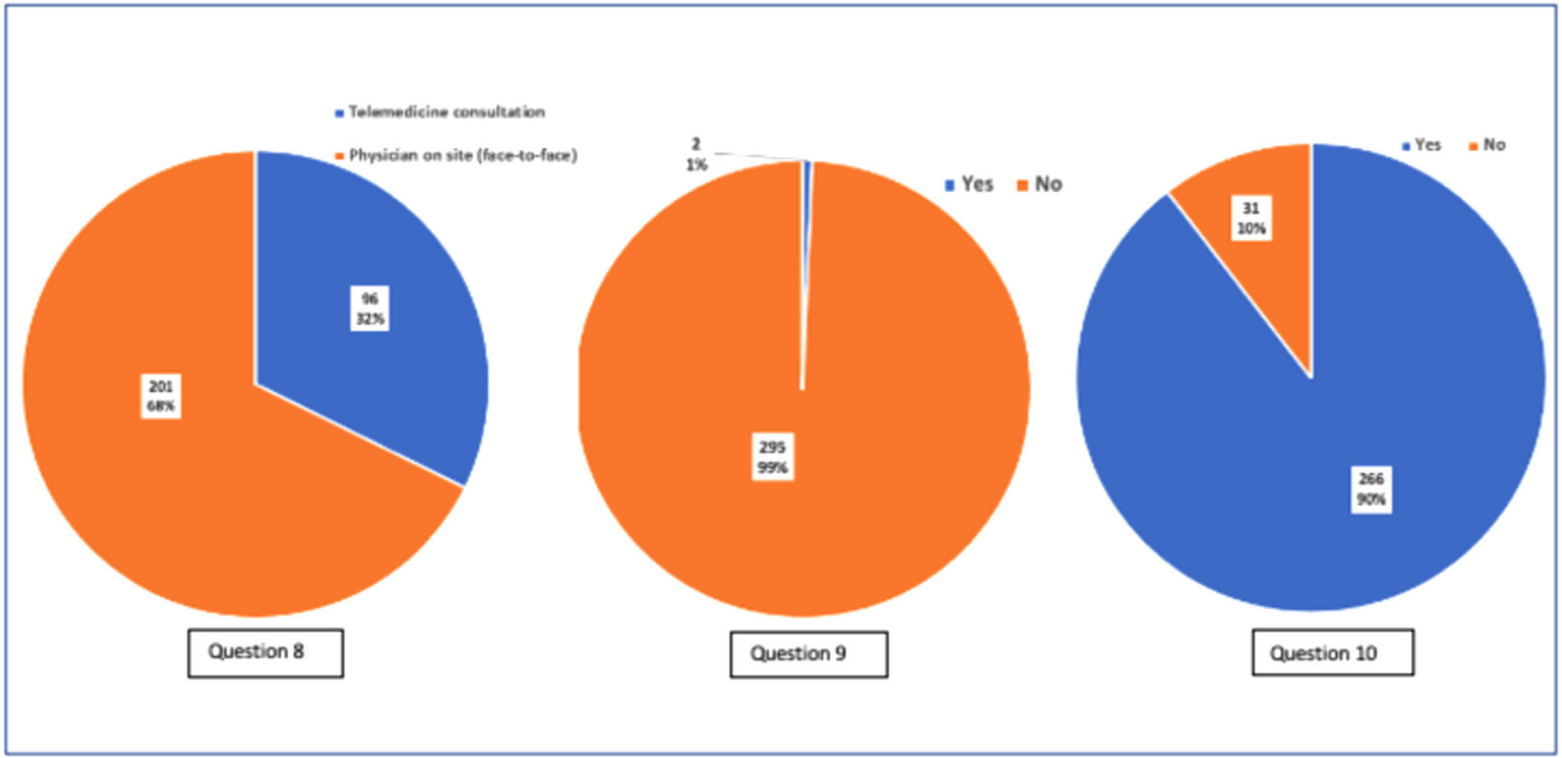
Patients feedback and satisfaction questions 8–10

We asked patients to rate their telemedicine experiences on a scale of 1 to 6, as demonstrated in Figures 1 and 2. Unsurprisingly, most of our cohort (99.3%) had never experienced a telemedicine consultation before. However, once exposed to the telemedicine method, most patients (42%) agreed that this form of consultation improved their confidence in their cancer care center. This was also reflected as, collectively, more than 80% somewhat to strongly agreed they were satisfied with the quality‐of‐care teleconsultations provided. In addition, when asked if telemedicine met their care needs, 87% of patients collectively somewhat strongly agreed that it did.

It seems evident that the level of communication through telemedicine was not compromised as most patients' felt that they could freely talk over the phone, with 89% somewhat to strongly agreeing collectively. This was also reflected through further questioning, as the majority also agreed that they could understand recommendations made by their physician over the phone (90% somewhat to strongly agreed). In addition, participants were generally satisfied with the quality of sound provided through teleconsultations, with 58% strongly agreeing.

Although overall, 88% somewhat to strongly agreed with their telemedicine experience, we found that if given a choice, most of the participants would prefer face‐to‐face consultations over teleconsultations (68% vs. 32%, respectively). However, the majority would still be willing to participate in future telemedicine consultations (90% vs. 10%, respectively).

## 4 DISCUSSION

**Figure 3:**
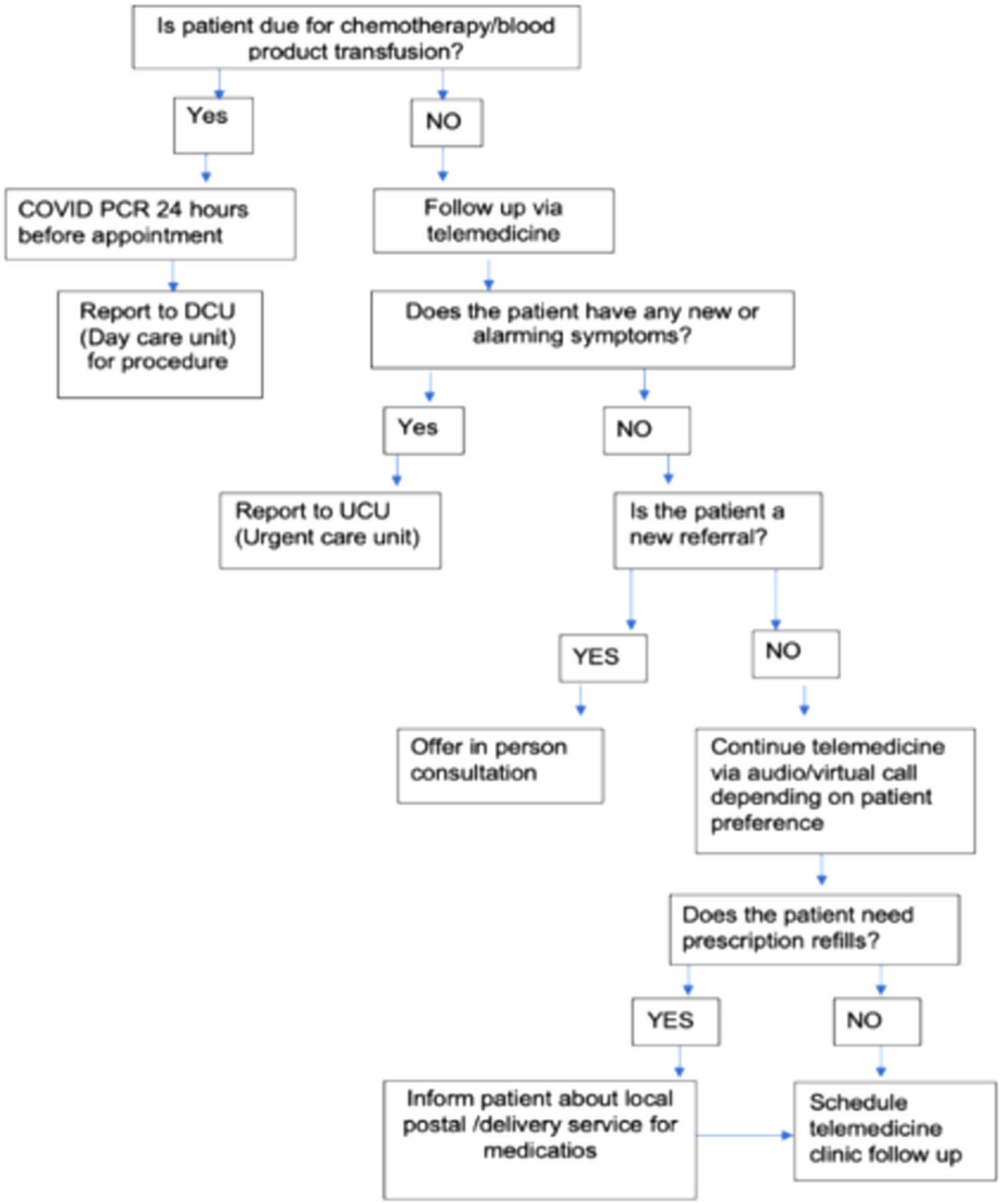
Workflow for conducting telemedicine clinics

This pandemic has set in motion a fleeting utilization of telemedicine in cancer care. With the best of our knowledge this is the first review regarding patients' satisfaction of telemedicine in Qatar. Incorporating a practical pathway approach to telemedicine, into our routine health care system needs careful planning. We proposed an outpatient workflow chart (Figure 3) that can be adapted across different cancer center systems. Many cancer patients showed a positive attitude towards telemedicine. We identified that, through their experience, 87% of respondents somewhat to strongly agreed that telemedicine was able to meet their care of needs.

These results are mirrored in some of the available literature, including a cross‐sectional study conducted in Canada by Gondal et al, in which out of 165 patients, 90% felt their needs were met, and 91% felt telemedicine improved their quality of care.^35^ Comparably, in a study conducted in Israel by Hasson et al, which investigated perceptions and perspectives of 172 cancer patients during the COVID‐19 pandemic, we see similarities as 82% of their patients felt their needs were provided for during teleconsultations.^22^ Our analysis also showed that 89% of respondents felt they could talk freely through telemedicine, which is similar to Hasson et al.^22^ who showed that 97% of their patients could describe and appropriately express their physical and mental wellbeing over telemedicine. Another descriptive cross‐sectional study conducted in Turkey showed that 92.8% of patients felt their needs were met via telemedicine with 93% feeling their queries had been solved over the phone or video without having to travel to their cancer center.^32^

It is important to note that although most of the respondents in our study accepted telemedicine, when it came down to their preference if given a choice, 68% preferred in‐person visitations. This was echoed by Wehrle et al.^20^ who conducted a study in the United States exploring physician and patient attitudes towards telemedicine during the pandemic; out of 374 patients, 68.2% of respondents preferred in‐person visits during the pandemic, and 80.4% even said that they would prefer in‐person clinics after COVID19 ceased.

Wehrle et al.^20^ rationalized this result by proposing that patients viewed contracting COVID 19 as less threatening than their malignancy; therefore, being physically seen and examined by a doctor was a necessary risk.

Although our study doesn't address reasons for preference to in‐person visits, we can speculate that one reason could be the quality of sound which was only accepted in 58% of our patients as well as lack of nonverbal communication. It would be interesting to see how additionally providing video interviews would improve overall satisfaction when considering factors such as hearing impairment and elderly age. We can see in a similar study to ours by Shaverdian et al, that telemedicine survey responses varied between telephone versus audiovisual visitations. Interestingly, when it came to factors associated with a preference for in‐person visitation, patients who used telephone‐only visitations were two times more likely to report that their understanding of the treatment plan would have been better with an in‐person visit. They justify these findings by reflecting on multiple studies that show nonverbal communication between physicians and patients leads to a higher patient satisfaction and quality of care.^36^

According to our study, given the opportunity to experience telemedicine again, 90% were willing to do so. Similarly, a study done in the United Kingdom by Smrke et al.^21^ showed that 80% out of 283 patients indicated they would like future telemedicine appointments if given the option. Although our study does not explore the reasoning behind positive responses or patient satisfaction, Smrke et al.^21^ explored possible reasons, including a patient preference for telemedicine as it was more cost‐effective with less travel time.

As our study is a qualitative survey analysis it is therefore subjected to confounders. There is a lack of categorization in accordance with the disease severity, duration of illness, and any disease‐related complications. One of the major disadvantages was the way we conducted our survey questionnaire. Factors such as the tone of the physicians' voice while reading the survey may have played as bias on patients' responses. Another downside is that physicians may have explained each value in the likert score system differently, which also could have created a wide variability of responses. To combat these potential bias influences, each patient could have been sent a virtual copy of the survey questionnaire. Our study also included diseases which varied in severity, responses may have been misleading in patients who were actively suffering from cancer complications. It is also possible that patients with predominately negative feedback were those with more disease severity and underlying depression, which is a major concern in cancer patients therefore affected such qualitative analytic studies.^37^

## 5 CONCLUSION

Telemedicine is a revolutionary system that has pioneered a new era for delivering patient care. Our study called attention to similar precedencies as well as disadvantages which mirrored the available literature on telemedicine use during the pandemic. Communication workshops on telemedicine among physicians and education for patients could further improve this healthcare delivery system. In our opinion, it is a perspective that can deliver optimum outpatient care to an eligible group of cancer patients or those who opt for it over in‐person visits. We do acknowledge that developing regions may be at disadvantage, due to lack of access in advanced technology among many other factors. Therefore, we encourage further tailored studies that can anticipate and overcome obstacles in the developing world. This will allow us to readily assess telemedicine's overall reliability on a global scale. As health care professionals, we should look to implementing patient and physician education so that we may expand the telehealth approach in the future.

## AUTHOR CONTRIBUTIONS

**Israa Elhakeem**: Data curation; methodology; supervision; validation; writing–original draft; writing–review and editing. **Phool Iqbal**: Data curation; methodology; supervision; validation; writing–original draft; writing–review and editing. **Abdulqadir J. Nashwan**: Conceptualization; data curation; methodology; validation. **Muhammad Abubakar**: Data curation; methodology; validation. **Arsalan T. Jawad**: Data curation; methodology; validation. **Mousa A. AlHiyari**: Data curation; methodology; validation. **Prem Chandra**: Data curation; methodology; supervision; formal analysis. **Maab A. Osman**: Data curation; methodology; validation. **Sara S. Mohamad**: Data curation; methodology; validation. **Mohammed Alkhatib**: Data curation; methodology; validation. **Mohamed A. Yassin**: Conceptualization; data curation; methodology; validation; writing–original draft. All authors have read and approved the final version of the manuscript.

## CONFLICT OF INTEREST

The authors declare no conflict of interest. Note: Abdulqadir J. Nashwan is an Editorial Board member of Health Science Reports and co‐authorco‐author of this article. He is excluded from editorial decision‐making related to the acceptance of this article for publication in the journal.

## ETHICS STATEMENT

The study was exempted by the Medical Research Center (MRC)—Institutional Review Board (IRB) at Hamad Medical Corporation (MRC‐01‐20‐929). The study has been conducted per the ethical standards noted in the 1964 Declaration of Helsinki and its later amendments or comparable ethical standards. Verbal consent was obtained from the participants.

## TRANSPARENCY STATEMENT

The lead author (Dr. Israa Elhakeem) affirms that this manuscript is an honest, accurate, and transparent account of the study being reported; that no important aspects of the study have been omitted; and that any discrepancies from the study as planned (and, if relevant, registered) have been explained.

## Data Availability

All data generated during this study are included in this published article. [Dr. Israa Elhakeem] had full access to all of the data in this study and takes complete responsibility for the integrity of the data and the accuracy of the data analysis.
